# Antioxidant and Neuroprotective Activities of *Hyptis suaveolens* (L.) Poit. Against Oxidative Stress-Induced Neurotoxicity

**DOI:** 10.1007/s10571-013-0016-7

**Published:** 2014-01-14

**Authors:** Hadi Ghaffari, Behrouz Jalali Ghassam, S. Chandra Nayaka, K. Ramachandra Kini, H. S. Prakash

**Affiliations:** Department of Studies in Biotechnology, University of Mysore, Mysore, 570006 Karnataka India

**Keywords:** *Hyptis suaveolens*, Antioxidant activity, Neuroprotective activity, DPPH, ABTS, FRAP

## Abstract

The present study was carried out to investigate the antioxidant and neuroprotective effects of *Hyptis suaveolens* methanol extract (HSME) using various in vitro systems. The total phenol and flavonoids contents of the HSME were quantified by colorimetric methods. The HSME extract exhibited potent antioxidant activity as determined by 2,20-azino-bis(3-ethylbenzothiazoline-6-sulfonic acid) diammonium salt, 2,2-diphenyl-1-picrylhydrazyl, and ferric reducing antioxidant power assays. The neuroprotective activity of HSME was determined on mouse N2A neuroblastoma cells using 3-(4,5-dimethylthiazol-2-yl)-2,5-diphenyltetrazolium bromide, lactate dehydrogenase, intracellular ROS assays, and upregulation of brain neuronal markers at genetic level. The N2A cells were pretreated with different concentrations (0.5, 1, 1.5, and 2 mg/ml) of the extract and then exposed to H_2_O_2_ to induce oxidative stress and neurotoxicity. The survival of the cells treated with different concentrations of HSME and H_2_O_2_ increased as compared to cells exposed only to H_2_O_2_ (47.3 %) (*p* < 0.05). The HSME also dose-dependently reduced LDH leakage and intracellular ROS production (*p* < 0.05). Pretreatment with HSME promotes the upregulation of tyrosine hydroxylase (2.41-fold, *p* < 0.05), and brain-derived neurotrophic factor genes (2.15-fold, *p* < 0.05) against H_2_O_2_-induced cytotoxicity in N2A cells. Moreover, the HSME showed antioxidant activity and decreased neurotoxicity. These observations suggest that HSME have marked antioxidant and neuroprotective activities.

## Introduction

Bioactive natural compounds commonly found in vegetables, fruits, herbs, and other plants have been shown to have possible health benefits with antioxidant, atherosclerosis, antimutagenic, and angiogenesis inhibitory activities (Cao and Cao 1999; Geleijnse et al. 1999; Yen et al. 2002). Interestingly, many plants are known to contain large amounts of phenolic and flavonoid antioxidants other than well-known vitamin C, vitamin E, carotenoids, quercetin, kaempferol, and isorhamnetin. Phenolic antioxidants in herbs are mainly composed of phenolic acids, flavonoids, and catechins. Some phenolic compounds in herbs could quench lipid peroxidation, prevent DNA oxidative damage, and scavenge reactive oxygen species (ROS), such as superoxide, hydrogen peroxide, and hydroxyl radicals (Cao and Cao 1999; Kahkonen et al. 1999).

ROS play an important role in oxidative damage to cellular compartment which leads to cell injury and death. This phenomenon is also responsible for various chronic diseases like coronary heart disease, carcinogenesis, and other health problems related to advancing age (Marnett 2000). Consequently, increased antioxidant intake in humans through diet is an important way to minimize such oxidative damage. The commercially available synthetic antioxidants are known to exhibit serious toxicity. Hence, there are efforts to search for natural, economical, and effective antioxidants. Many plant species in diverse regions of the world have been screened for their antioxidant activity (Yingming et al. 2004). Due to their biodiversity, the active principles differ from plant to plant and they produce definite physiological actions on human body.

The plant *Hyptis suaveolens* (L.) Poit commonly known as “Wilayati tulsi” belongs to the family Lamiaceae and is an ethnobotanically important medicinal plant. The plant has been considered as a weed, distributed throughout the tropics and subtropics. Almost all parts of this plant are being used in traditional medicine to treat various diseases. In Indian traditional medicine, the leaves are used as stimulant, carminative, and in treatment of stomach ache. Both leaves and twigs are considered to exhibit antispasmodic activity and are used as source of anti-inflammatory, antioxidant activity, and antifertility agents and also as antiseptic in burns, wounds, and various skin complaints (Shirwaikar et al. 2003; Gavani and Paarakh 2008; Shenoy et al. 2009) as well as methanol and aqueous extracts have shown hepatoprotective and cytoprotective activities (Ghaffari et al. 2012; Babalola et al. 2011); moreover, Suaveolol isolated compound of this plant showed gastroprotection (Vera-Arzave et al. 2012) .

Chemically, the plant contains several constituents such as carbohydrates, tannins, phenols, saponins, steroids, alkaloids, and glycosides, which are responsible for the medicinal activities. The genus *Hyptis* possesses a diverse range of biological activities which have been attributed to essential oil obtained by hydrodistillation of the leaves of *H. suaveolens* (Peerzada 1997). Based on the traditional knowledge and recent pharmacological studies, the objective of the present study was to investigate the antioxidant and neuroprotective effects of methanol extract of *H. suaveolens* by various in vitro assays.

## Materials and Methods

### Chemicals

2.4.6-Tripyridyl-s-Triazine (TPTZ), 1,1-diphenyl, 2-picrylhydrazyl radical (DPPH), 2,2-Azinobis (3-ethyl benzothiazoline-6-sulfonic acid) (ABTS), Eagle’s minimum essential medium, trypsin (0.1 %), MTT [3-(4,5-dimethylthiazol-2-yl)-2, 5-diphenyltetrazolium bromide], fetal calf serum, DCFH-DA (2′,7′-dichlorofluorescein-diacetate), HS-RT- PCR kit, and RNA- isolation kit were purchased from Sigma, USA. Cyber green master mix (2X) was purchased from Qiagen (Gamb, Germany). Ascorbic acid, 6-hydroxy-2,5,7,8-tetramethylchroman-2-carboxylic acid (trolox), ferric chloride, hydrogen peroxide, and potassium persulfate were purchased from Himedia, Mumbai, India. Lactate dehydrogenase kit was purchased from Agappe diagnostics, Kerela, India.

### Extraction of Plant Sample

Fresh aerial parts of *H. suaveolens* were collected in the month of February 2012 from Mysore, India and were authenticated by a plant taxonomist. Healthy plants were screened and thoroughly washed to remove adhering dust and shade-dried. The dried plants were pulverized in a mechanical grinder and 50 g of coarse powder was serially extracted with hexane, ethyl acetate, and methanol using Soxhlet apparatus. The extracted solvent was filtered and evaporated to dryness in a flash evaporator and the dried extracts were collected and stored in refrigerator for further studies.

### Estimation of Total Phenolic Content

The total phenolic content of HSME was determined using the Folin–Ciocalteu reagent method (Lister and Wilson 2001). To 50 μl of each extract, 2.5 ml of Folin–Ciocalteu reagent (1/10 dilution) and 2 ml of 7.5 % Na_2_CO_3_ (w/v) were added and mixed well. The blend was incubated at 45 °C for 15 min. The absorbances of all samples were measured at 765 nm with Na_2_CO_3_ solution (2 ml of 7.5 % Na_2_CO_3_ in 2.55 ml of distilled water) as blank. The results were expressed as μg of gallic acid equivalence (GAE)/mg dry weight extract.

### Estimation of Flavonoid Content

The flavonoid content was determined by Aluminum chloride (AlCl_3_) method (Chang et al. 2002). The reaction mixture consisted of 1.0 ml extract, 0.5 ml AlCl_3_ (1.2 %), and 0.5 ml potassium acetate (120 mM). The mixture was allowed to stand for 30 min at room temperature and the absorbance of the reaction mixture was measured at 415 nm. The flavonoid content is expressed in terms of μg quercetin equivalent/mg dry weight extract.

### DPPH Free Radical Scavenging Activity

DPPH free radical scavenging activity was determined according to the method of Sultanova et al. (2001). Different concentrations of HSME were prepared, while the concentration of DPPH was 300 μM in the reaction mixture. The reaction mixture contained 5 μl of test samples and 95 μl of DPPH in methanol. These reaction mixtures were taken in 96-well microtiter plates and incubated at 37 °C for 30 min. The absorbance was measured at 517 nm. Percent radical scavenging activity upon sample treatment was determined by comparison with a methanol-treated control. All determinations were performed in triplicates. Ascorbic acid was used as positive control.

$${\text{Radical scavenging }}\left( \% \right) = \left[ {\left( {A_{\text{C}} {-}A_{\text{S}} } \right)/A_{\text{C}} } \right] \times 100$$

where *A*_C_ is the absorbance of the control (methanol-treated) and *A*_S_ is the absorbance of the antioxidants in the crude extract and standard.

### ABTS (2,2′-azino-bis 3-ethylbenzthiazoline-6-sulfonic) Radical Cation Decolorization Assay

For ABTS assay also two different concentrations have been tried. The scavenging activity of ABTS^•**+**^ was measured (ABTS^•**+**^assay) according to the method described by Re et al. (1999). ABTS was dissolved in water to 7 mM concentration. ABTS radical cation (ABTS^•**+**^) was produced by reacting ABTS stock solution with 2.45 mM potassium persulfate (final concentration) and allowing the mixture to stand in the dark at room temperature for 12–16 h before use. The ABTS^•**+**^ solution was diluted with ethanol/methanol to an absorbance of 0.70 (± 0.02) at 734 nm and equilibrated at 30 °C. Methanol was used as a negative control. After addition of 1.0 ml of diluted ABTS^•**+**^ solution (A734 nm = 0.700 ± 0.020) to 10 μl of HSME and 6-hydroxy-2,5,7,8-tetramethylchroman-2-carboxylic acid standard in methanol the absorbance was taken at 734 nm, exactly one min after initial mixing and up to six min using the spectrophotometer. Appropriate solvent blanks were run in each assay. All determinations were carried out at least three times, and in triplicates, on each occasion and at each separate concentration of the standard and samples. The ABTS^•**+**^ scavenging capacity of extract compared with 6-hydroxy-2,5,7,8-tetramethylchroman-2-carboxylic acid and percentage inhibition was calculated, same as DPPH assay.

### Ferric Reducing Antioxidant Power Assay (FRAP)

This assay has been described by Benzie and Strain (1996). In fact, ferric reducing antioxidant power (FRAP) assay measures the change in absorbance at 593 nm due to the formation of a blue-colored complex of ferrous ion (Fe^2+^) and 2,4,6-tripyridyl-s-triazine (TPTZ). Prior to this, colorless ferric ion (Fe3 +) was oxidized to ferrous ion (Fe^2+^) by the action of electron-donating antioxidants. Freshly prepared FRAP reagent was kept at 37 °C in a water bath. This reagent was prepared by mixing 10 mM of TPTZ in 40 mM HCl, 20 mM FeCl_3_, and 0.3 M acetate buffer (pH 3.6) in the ratio of 1:1:10. An aliquot of 25 μl of HSME was added to 475 μl of FRAP reagent. The mixture was incubated at 37 °C for 30 min. Absorbance was read at 593 nm using a UV–Vis Spectrophotometer. The reducing ability was calculated with reference to the reaction given by FeSo_4_·7H_2_O. The values were expressed as mM FeSO_4_/g dry weight of plant extracts.

### Cell Culture and Treatments

Mouse Neuroblastoma cell line (N2A) was obtained from national centre for cell science (Pune, India). Cells were grown in 25-cm^2^ flasks with loosened caps, containing Ham’s F12 supplemented with 10 % fetal bovine serum and 2 mM l-glutamine at 37 °C (NuAire, Plymouth, MN, USA) in an atmosphere of humidified 5 % CO_2_. To check possible toxic effects, the cells were treated with various concentrations of HSME (0.1–2 mg/ml) for 24 h. To induce oxidative stress, the cells were exposed to freshly prepared 100 μM H_2_O_2_ for 24 h. Cells were pretreated with HSME for 2 h before the addition of 100 μM H_2_O_2_. After 24 h, the cell viability was determined by MTT assays.

### Analysis of Cell Viability Using MTT Assay

MTT assay was performed as described by Mosmann with some modifications (Mosmann 1983). Based on the preliminary observations, cells in the exponential phase were seeded onto 96-well plates (10 × 10^4^ cells/well), allowed to adhere for 24 h, and treated with various concentrations of HSME (0.1–1 mg/ml) along with 100 μM H_2_O_2_. The medium following treatments was removed, cells were washed with PBS, and 100 μl of the MTT (5 mg/ml) was added to each well. After 4 h of incubation, the solution was removed and 100 μl of DMSO was added to each well. After 10 min, the wells were read at 570 nm on an ELISA reader. The viability (%) was calculated as follows:

$${\text{Viability }} \% = \frac{{{\text{Average of test wells O}}.{\text{D}}{-}{\text{Average of blank wells O}}.{\text{D}}}}{{{\text{Average of control wells O}}.{\text{D}}{-}{\text{Average of blank wells O}}.{\text{D}}}} \times 100$$

### Lactate Dehydrogenase (LDH) Release Assay

LDH is a marker enzyme for cell degeneration. The amount of LDH was measured using LDH estimation kit (Agappe diagnostics) according to the manufacturers’ instructions. In brief, the N2A cells were plated at a density of 5 × 10^4^ cells/well in 24-well plates; following 24 h, the cells were treated with different concentrations of HSME for 2 h. After pretreatment the cells were treated with 100 μM H_2_O_2_ for 24 h. The cells were precipitated by centrifugation at 2,500 rpm for 5 min at RT and the supernatant was used to measure the amount of released LDH.

### Observations of Morphological Changes

The cells were seeded in Petri dishes (1 × 10^5^ cells) and then treated with different concentrations of extracts and exposed to H_2_O_2_. The cellular morphology was observed and photographed using a phase contrast microscope (Zeiss, Germany) equipped with Cool SNAP^®^ Pro color digital camera.

### Measurement of Intracellular ROS

The intracellular ROS was estimated to measure oxidative stress induced by H_2_O_2_ using oxidation-sensitive dye DCFH-DA (Wang and Joseph 1999). The assay is based on the principle that the nonfluorescent fluorescein DCFH-DA derivatives will emit fluorescence after being oxidized by the radicals generated by H_2_O_2_. The emitted fluorescence is directly proportional to the concentration of generated radicals. The nonionic, nonpolar DCFH-DA crosses cell membranes and is hydrolyzed enzymatically by intracellular esterases to nonfluorescent DCFH which is oxidized to highly fluorescent dichlorofluorescein (DCF) in the presence of ROS (Lebel et al. 1992). Therefore, the intracellular DCF fluorescence can be used as an index to quantify the overall oxidative stress generated in the cells. The cells were plated at a density of 5 × 10^4^ cells/well in 24-well plates; after 24 h, cells were treated with different concentrations of HSME and exposed to H_2_O_2_. After treatments, 5 mg/ml DCFH-DA was added to the cells and incubated for 30 min. Then the cells were washed twice with PBS and the fluorescence was detected at an excitation wavelength of 485 nm and an emission wavelength of 535 nm using Hidex plate chameleon™ V (Finland).

### Effects of HSME on H_2_O_2_ Altered Brain Neuronal Marker Gene Expression

N2A cells were cultured in 75 cm^2^ flasks (1 × 10^6^), pretreated with 1 mg/ml HSME, and exposed to H_2_O_2_ for 24 h. Total cellular RNA was isolated with a commercial RNA-isolation kit followed by manufacturer’s instructions (Sigma, St Louis, MO, USA). Equal amounts (2 μg) of RNA were primed with oligo (dT) primers and reverse-transcribed using a HS-RT-PCR kit (Sigma, St Louis, MO, USA). The gene expression of neuronal biomarkers were analyzed by quantitative real-time PCR (RT-PCR) using SYBR Green I Mastermix (Qiagen, Gambh, Germany) total volume of 30 μl containing appropriate target primers of Brain-derived neurotrophic factor (BDNF F-ATGACCATCCTTTTCCTTACT-, BDNF R-GCCACCTTGTCCTCGGAT-) and Tyrosine hydroxylase (TH F-GAGGAGAAGGAGGGGAAG-, TH R-ACTCAAACACCTTCACAGCT-) followed by manufacturer’s instructions using a Roche Light Cycler 480. Each sample was assayed in duplicates and the relative gene expression was quantified using ΔΔCT method by normalizing with β-2 myoglobulin as a housekeeping gene.

### Statistical Analysis

Data are presented as mean ± standard deviation (SD). The significance of the difference from the respective controls for each experimental test condition was assayed by using “Student’s t-test” for each experiment as *p* < 0.05.

## Results

### Total Phenol and Flavonoids Content

The results of quantitative phytochemical analysis of total phenol and flavonoid content of the HSME are shown in Table 1. The total phenolic and flavonoids contents are expressed as μg gallic acid equivalents/mg of extract and μg quercetin equivalents/mg of extract, respectively. HSME contains higher phenolic content compared to flavonoids and the phenolic contents are 2.6-folds more than flavonoids. Hence, the phenolic compounds in this plant might be bioactive compounds.

**Table 1:** Total phenol, flavonoid contents and FRAP activity in *H. suaveolens* methanol extract (HSME)

Experiment	HSME
Total phenols content (μg gallic acid equivalents/mg extract)	74.56 ± 1.33
Flavonoids content (μg quercetin equivalents/mg extract)	28.58 ± 1.74
FRAP activity (mM FeSO4/g dry weight of extract)	1.76 ± 0.15

### Radical Scavenging Activity

**Fig. 1:**
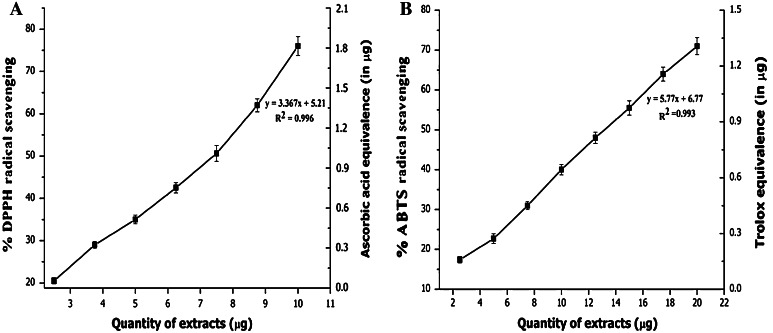
DPPH (**a**) and ABTS (**b**) radical scavenging activity of *H. suaveolens* methanol extract (HSME) with Ascorbic acid and 6-hydroxy-2,5,7,8-tetramethylchroman-2-carboxylic acid (trolox) equivalence (in μg). HSME exhibited antioxidant activity with an IC_50_ of 7.49 and 12.43 μg, respectively. Data expressed as mean ± SD along with regression and correlation (*n* = 3)

HSME showed a concentration-dependent scavenging of DPPH radicals and was found to be an active radical scavenger with IC_50_ value of 7.49 μg. The activity was compared to ascorbic acid which is employed as the standard and results were plotted against ascorbic acid equivalence in μg (Fig. 1a). Proton radical scavenging is an important attribute of antioxidants. HSME was also an effective scavenger of ABTS radicals and the activity was comparable to that of 6-hydroxy-2,5,7,8-tetramethylchroman-2-carboxylic acid (Fig. 1b). HSME showed antioxidant activity in ABTS assay with IC_50_ value of 12.43 μg. The higher concentrations of the extracts were more effective in quenching free radicals in the system. The scavenging of the ABTS radical by the extracts was found to be much higher than that of DPPH radical.

### Ferric Reducing Ability of HSME

FRAP assay is based on a oxidation/reduction-linked reaction, whereby antioxidants present in plant extracts act as reductants while ferric ions in reagents serve as oxidants. Reduction of ferric–TPTZ to ferrous–TPTZ complex forms an intense blue color with maximum absorption at 593 nm, which is related to the amount of antioxidants in the sample. The ferric reducing ability of HSME is shown in Table 1.

### Effect of Different Concentrations of HSME on Cell Viability in H_2_O_2_-Induced Oxidative Stress on N2A Cells

**Fig. 2:**
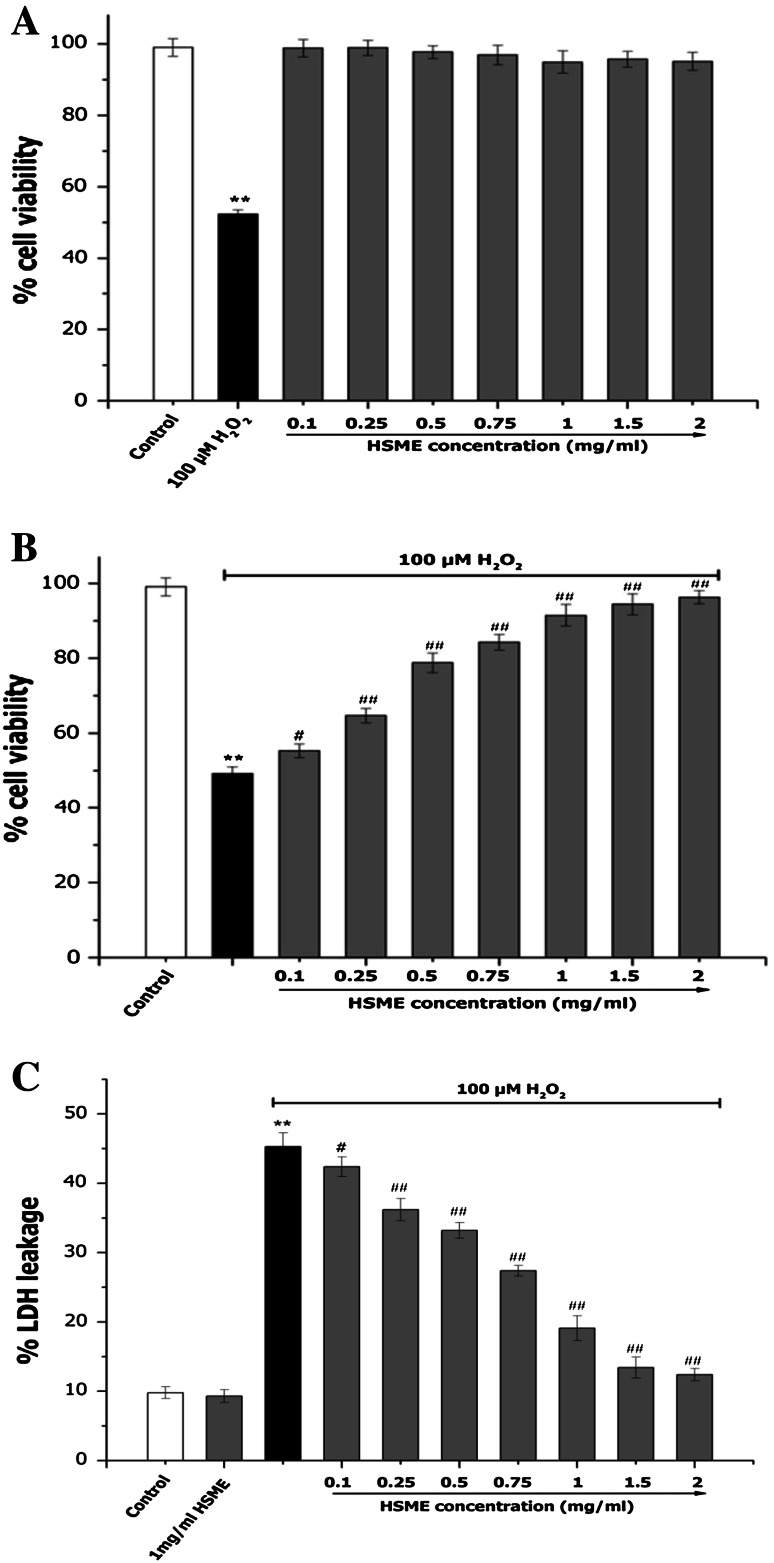
Effect of HSME on N2A cell viability (**a**), H_2_O_2_-induced oxidative stress (**b**) and LDH leakage (**c**). **a** N2A cells were exposed to various concentrations of HSME and 100 μM H_2_O_2._ **b** N2A cells treated with various concentrations of HSME were exposed to 100 μM H_2_O_2_ for 24 h. Cell proliferation was determination by MTT assay. **c** N2A cells treated with various concentrations of HSME were exposed to 100 μM H_2_O_2_ for 24 h. LDH levels in the cells supernatant was determined using ELISA. Results represent mean ± SD (*n* = 3) for each concentration. ** = *p* < 0.05 compared to control, ^#^ = *p* < 0.05 compared to H_2_O_2_ group. ^##^ = *p* < 0.01 compared to H_2_O_2_ group

The exposure of different concentrations of HSME (0.1–2 mg/ml) on cell viability in N2A cells for 24 h did not alter the viability (Fig. 2a). The cell viability of N2A cells was increased with the treatment of different concentrations of HSME. The treatment with 100 μM H2O2 on N2A cells for 24 h showed cell toxicity. Treatments with different concentrations (0.1–2 mg/ml) of HSME for 2 h prior to the addition of 100 μM H_2_O_2_ for 24 h induced a dose-dependent increase of cell viability. Moreover, at 2 mg/ml of HSME H_2_O_2_-induced stress was completely neutralized demonstrating the effectiveness of HSME in preventing oxidative stress to N2A cells (Fig. 2b).

LDH release is an indirect measure of cell viability. To further investigate the protective effect of HSME, the release of LDH was measured in the presence and absence of H_2_O_2_ (Fig. 2c). When N2A cells were treated with 2 mg/ml HSME, the levels of LDH remained identical to control indicating the non-toxic nature of HSME. However, upon exposure to 100 μM H_2_O_2_ for 24 h, the cell supernatant contained increased LDH compared to control indicating the cytotoxicity of H_2_O_2_. On the contrary, cells pretreated with 0.1–2 mg/ml HSME demonstrated decreased amounts of LDH leakage signifying dose-dependent protective effect of HSME against H_2_O_2_-induced cytotoxicity (Fig. 2c).

### Effect of HSME on Morphological Changes and Decreased Intracellular ROS Against Exposure to H_2_O_2_

**Fig. 3:**
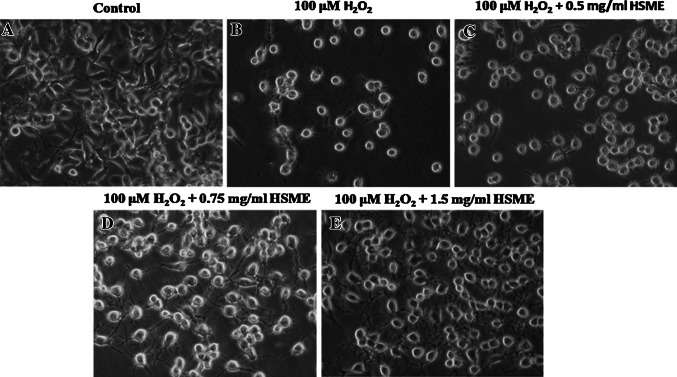
The effects of HSME in H_2_O_2_-induced cell morphological changes. **a** Untreated, **b** N2A cells exposed to H_2_O_2_, **c**, **d** and **e** N2A cells pretreated with various concentrations of HSME (0.5, 0.75, and 1.5 mg/ml) for 2 h before exposed to 100 μM H_2_O_2_ for 24 h

**Fig. 4:**
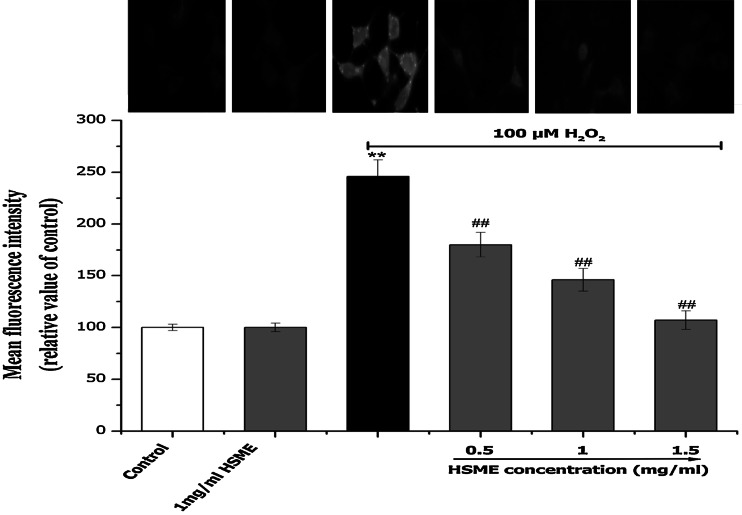
Effect of different concentrations of HSME on the intracellular ROS formation in N2A cells. Intracellular ROS levels were measured by the fluorescent probe DCFH-DA after 24 h exposed to H_2_O_2_ pretreated with HSME for 2 h. The fluorescence intensity was expressed as relative value of control (% of control). Data are presented as mean ± SD from three independent experiments. ** = *p* < 0.05 compared to control. ^##^ = *p* < 0.05 compared to H_2_O_2_ group

As shown in Fig. 3, pretreatment of N2A cells with different concentrations (0.5–1.5 mg/ml) of HSME exposed to H_2_O_2_ reduced the toxicity and change of cells morphology as compared to H_2_O_2_-exposed cells. Intracellular ROS is another assay to measure the cell viability. To verify the contribution of mitochondria-derived ROS to H_2_O_2_-induced oxidative damage and lesions, we compared H_2_O_2_-induced intracellular oxidant production in N2A cells. The overall ROS level was determined using DCFH-DA, a cellular membrane-permeable nonfluorescent probe that can be irreversibly oxidized by intracellular ROS into a green fluorescent product, DCF. N2A cells pretreated to various concentrations of HSME (0.5–1.5 mg/ml) for 2 h and exposed to 100 μM H_2_O_2_ for 24 h did not affect the cell viability. However, exposure of cells to 100 μM H_2_O_2_ alone caused significant oxidative stress and cell death (Fig. 4).

### HSME Stimulates Enhanced Expression of Genes Encoding Brain Neuronal Markers in N2A Cells

**Fig. 5:**
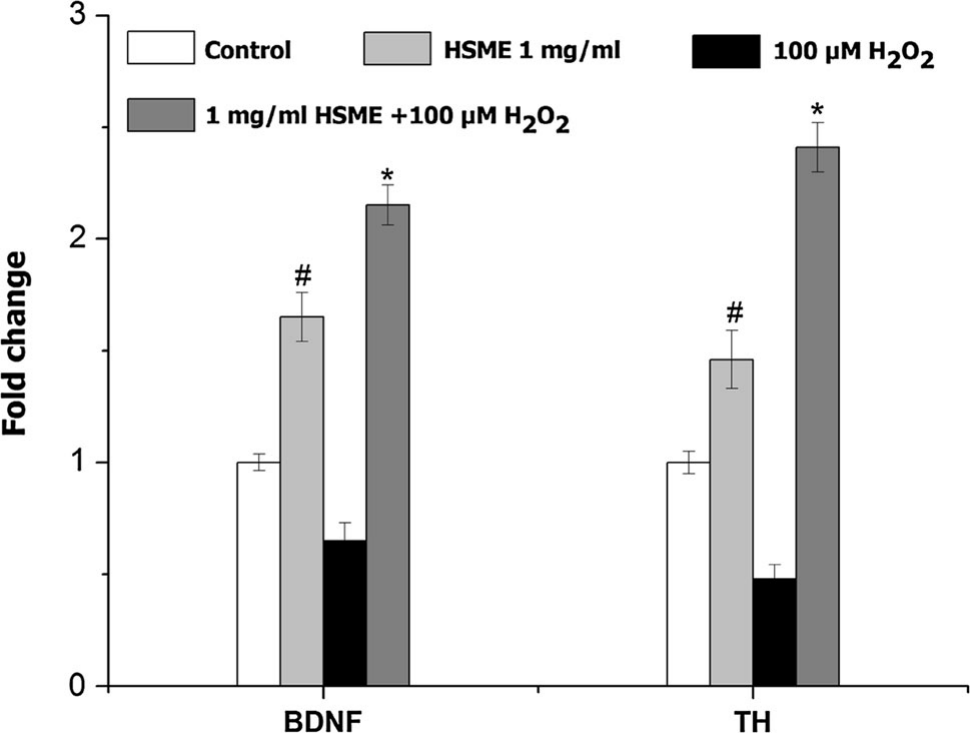
Real-time PCR for quantification of brain neuronal markers expression levels following exposure to HSME, H_2_O_2_, and HSME + H_2_O_2_. The fold change was calculated based on normalization with β-2 myoglobulin gene expression. The analysis was performed with Light Cycler and relative quantification software. Each experiment was performed in triplicates. The data represent mean ± SD (*n* = 6). ^#^ = *p* < 0.05 compared to untreated control. * = *p* < 0.05 compared to H_2_O_2_ group. *BDNF*, brain-derived neurotrophic factor, *TH*, tyrosine hydroxylase

BDNF and TH play a major role in brain homeostasis by regulating the neurotransmitter metabolism. The gene response for BDNF and TH were monitored by quantitative real-time RT-PCR assay (Fig. 5). Cells pretreated with 1 mg/ml of HSME alone showed 1.65- and 1.46-folds increase in expression of BDNF and TH genes, respectively, (*p* < 0.05) compared to untreated control. However, treatment with 100 μM H_2_O_2_ alone resulted in decreased expression of the genes expression. Significantly, N2A cells pretreated with 1 mg/ml of HSME following exposure to 100 μM H_2_O_2_ displayed 2.15- and 2.41-folds increases in BDNF and TH expression levels, respectively, (*p* < 0.05) compared to H_2_O_2_ group.

## Discussion

The Folin–Ciocalteu assay, recognized as one of the standard antioxidant testing procedures (Prior et al. 2005), measures the level of total phenolics in natural products. Phenolic compounds are the major plant secondary metabolites with antioxidant activity. This activity is believed to be mainly due to their oxidation/reduction properties, which play an important role in adsorbing and neutralizing free radicals, quenching singlet and triplet oxygen, or decomposing peroxides (Long et al. 2000). In the present study, as part of analysis of chemical composition, total phenolics and flavonoids contents of *H. suaveolens* methanol extract (HSME) were determined. Results showed that phenolic compounds were present in considerable amount as compared to flavonoids content. The radical scavenging activity clearly showed a positive correlation between radical scavenging activity (decreased IC_50_ value) and increasing total phenol content.

The radical scavenging activity of HSME was studied by their ability to bleach the stable DPPH and ABTS free radicals, which provides information on the reactivity of compounds with a stable free radical (Badami et al. 2003). The results showed that HSME is effective in scavenging DPPH and ABTS radicals. Though the DPPH and ABTS radical scavenging abilities of the extract were less than those of Ascorbic acid and 6-hydroxy-2,5,7,8-tetramethylchroman-2-carboxylic acid and the study showed that the extract has scavenging or proton-donating ability and could serve as free radical scavenger, acting possibly as primary antioxidants. The DPPH radical scavenging assays revealed that the extracts might prevent reactive radical species from damaging biomolecules such as lipoproteins, polyunsaturated fatty acids, DNA, amino acids, proteins, and sugars in biological systems (Halliwell et al. 1995). Factors like stereo-selectivity of the radicals or the solubility of the extract in different testing systems have been reported to affect the capacity of extracts to react and quench different radicals and studies have shown that some compounds which have ABTS scavenging activity did not show DPPH scavenging activity (Wang et al. 1998; Yu et al. 2002). In ferric reducing/antioxidant power assay (FRAP), the antioxidant potentials of HSME was estimated from their ability to reduce Fe(III) complex to Fe(II). The result obtained from this assay supported the findings of the DPPH and ABTS assay and reconfirms the antioxidant potential of the HSME. Preliminary phytochemical analyses of the HSME also gave positive results for the presence of polyphenols and flavonoids which could be responsible for the antioxidant potential of this plant.

H_2_O_2_-induced cytotoxicity is the common method employed for the measurement of potential neuroprotective antioxidants (Chow et al. 2005; Fallarero et al. 2006; Garcia-Alonso et al. 2006). Most of antioxidants protect cells from massive oxidative stress by millimolar concentrations of H_2_O_2_. The N2A cells exposed to H_2_O_2_ were distinctively low in viability and prevented the gap junction intercellular communication. However, the cells pretreated with HSME increased cell viability and protected from H_2_O_2_-Induced oxidative stress. In this study, the morphological observation and the number of communicating cells treated with HSME were very similar to those of the untreated control cells. HSME protected the N2A cells from the H_2_O_2_-induced oxidative stress and maintained high level of cellular communication.

Moreover, useful information was obtained by monitoring LDH release into the media on the same samples. LDH is commonly utilized as an index of the integrity of cell membranes or necrosis in response to the oxidant burden (Mi and Zhang 2005). As shown in Fig. 2c, there is a marked increase of LDH release in H_2_O_2_-treated cells. However, this increase is markedly attenuated by the HSME treatment. In comparison with untreated control cells, the cell cultures pretreated with HSME displayed larger amounts of formazan after the MTT viability test and lower levels of LDH in the culture medium and decreased amount of intracellular ROS.

Brain-derived neurotrophic factor (BDNF) and tyrosine hydroxylase (TH) play a major role in neurotransmitter synthesis as well as brain functioning (Chen et al. 2003). The TH and BDNF expressions play a major role in the survival and maturation of dopaminergic neurons. The effect of H_2_O_2_-induced oxidative stress-mediated neuronal damage is well known. The downregulation of TH and AADC with simultaneous depletions of catecholamines in Parkinson’s disease has been reported by Ichinose et al. (1994). We observed (real-time PCR analysis) that N2A cells treated with HSME demonstrated enhanced expression of neuronal biomarker genes BDNF and TH (Fig. 5). Our studies are also in line with study on rosmarinus extract which contains rosmarinic acid as a potent inducer of TH and AADC against H_2_O_2_ stress in SH-SY5Y cells (Park et al. 2010). Choi et al. (2010) reported the elevated expression of TH and BDNF genes following treatment with Tripterygium extract in a similar model.

*H. suaveolens* methanol extract effectively prevented H_2_O_2_-induced toxicity which explains its health-promoting property. Previous studies with CCl_4_ and H_2_O_2_, an inducer of oxidative stress, also showed hepatoprotective and cytoprotective effects of *H. suaveolens* extracts (Ghaffari et al. 2012). Babalola et al. (2011) also reported the hepatoprotective activity of *H. suaveolens* aqueous extract against acetaminophen-induced oxidative stress in rabbit. The neuroprotective, hepatoprotective, and cytoprotective activities of *H. suaveolens* extract could be a result of free radical scavenging activity or boosting the antioxidant capacity of the body.

The present study showed the antioxidant and neuroprotective effects of HSME. The results reveal that HSME inhibits H_2_O_2_-induced neuronal death and ROS generation. Treatment of HSME can prevent H_2_O_2_-induced damage to N2A cells through antioxidant and protective gene upregulation. These data indicate that HSME may be employed to treat stress-induced neurodegeneration. However, further in vitro studies are necessary to better clarify its neuroprotective mechanism of action.
